# *Listeria monocytogenes* Virulence, Antimicrobial Resistance and Environmental Persistence: A Review

**DOI:** 10.3390/pathogens9070528

**Published:** 2020-06-30

**Authors:** Lavious Tapiwa Matereke, Anthony Ifeanyi Okoh

**Affiliations:** 1 SAMRC Microbial Water Quality Monitoring Centre, University of Fort Hare, Alice 5700, South Africa; 2 Applied and Environmental Microbiology Research Group, Department of Biochemistry and Microbiology, University of Fort Hare, Alice 5700, South Africa

**Keywords:** *Listeria monocytogenes*, virulence, biofilm, antimicrobial resistance, environmental stress

## Abstract

*Listeria monocytogenes* is a ubiquitous opportunistic pathogen responsible for the well-known listeriosis disease. This bacterium has become a common contaminant of food, threatening the food processing industry. Once consumed, the pathogen is capable of traversing epithelial barriers, cellular invasion, and intracellular replication through the modulation of virulence factors such as internalins and haemolysins. Mobile genetic elements (plasmids and transposons) and other sophisticated mechanisms are thought to contribute to the increasing antimicrobial resistance of *L. monocytogenes*. The environmental persistence of the pathogen is aided by its ability to withstand environmental stresses such as acidity, cold stress, osmotic stress, and oxidative stress. This review seeks to give an insight into *L. monocytogenes* biology, with emphasis on its virulence factors, antimicrobial resistance, and adaptations to environmental stresses.

## 1. Introduction

*Listeria* species are ubiquitous Gram-positive rods found in different environmental niches. Among the species in the genus *Listeria*, *Listeria monocytogenes* and *Listeria ivanovii*, are pathogenic, with the former being a human foodborne pathogen causing listeriosis and associated with other human ailments such as bacteremia, encephalitis, and sepsis [1]. *L. monocytogenes* was proven as the causative agent of listeriosis following an outbreak in 1989, but the pathogen had been detected as early as 1924 [2]. It is a psychrotolerant pathogen, capable of growing at different temperatures (1–45 °C), but the optimum temperature ranges from 30 to 37 °C [3]. The pathogen is proficient in switching between saprophytism and virulence, depending on the environmental setting [4].

Infection by *L. monocytogenes* results in either non-invasive gastrointestinal listeriosis among immunocompetent individuals or invasive listeriosis among immunocompromised people [5,6]. Invasive listeriosis leads to abortion in pregnant women and meningitis in immunocompromised individuals [7]. During the 2017–2018 listeriosis outbreak in South Africa, a 42% fatality rate was recorded, with more than 1000 laboratory-confirmed cases, and the most infected were infants and pregnant women [8]. Individuals on medications that minimize gastric acidity as well as chronic renal failure and cirrhosis patients are also at risk [9]. Also, *Listeria*-related urinary tract infections were reported in an incident wherein *L. monocytogenes* was detected in urine samples [10].

The major transmission route to humans is contaminated food, mainly ready-to-eat foods [11]. Irrigation waters and agricultural soils also harbor multidrug-resistant *L. monocytogenes* that is likely to be disseminated to agricultural fresh produce, posing a threat to food safety [12]. Poor hygiene practices and inadequate implementation of standard sanitation operating procedures (SSOPs) in the food processing industry have led to listeriosis outbreaks [13]. Furthermore, the pathogen easily forms biofilms and persister cells on surfaces, which may not be easily removed by standard sanitation protocols [14]. *L. monocytogenes* can be found concealed in difficult-to-clean tools such as slicers, food transport vehicles, and refrigeration units [14]. The pathogen also inhabits the gastrointestinal tract of humans and animals; hence, unhygienic practices may facilitate its dispersal through the contamination of food and equipment [2].

Adaptations of the pathogen to environmental stresses and pre-exposure adaptation to sublethal concentrations of antimicrobial agents have subsequently contributed to its antimicrobial resistance [15,16]. Therefore, understanding the principal mechanisms governing *L. monocytogenes* survival, virulence, antimicrobial resistance, and persistence in adverse environmental conditions is vital for the management and control of this pathogen, as well as the development of novel antimicrobial agents against it.

## 2. Virulence Factors

### 2.1. Hemolysins

Production of hemolysins by *Listeria* species was first demonstrated by Harvey and Faber in 1941 using *L. monocytogenes* [17]. It was later shown that this hemolysin is an ortholog of streptolysin O (SLO) from *Streptococcus pyogenes* and hence was named listeriolysin O (LLO) [18]. Encoded by the *hly* gene, listeriolysin O is a pH-controlled toxin that has been shown to promote infection from several niches within the host, mainly the extracellular environment, phagosome, and cytosol [19]. In the extracellular environment, LLO facilitates the internalization of *L. monocytogenes* into phagosomes by creating membrane perforations into the host cell [20]. LLO induces ion-permeable pores on the plasma membrane which allow calcium influx into the host cells, and this promotes the invasion of Hep-2 epithelial cells by *L. monocytogenes* [21]. LLO also mediates apoptosis of T cells, lymphocytes, and dendritic cells [22]. Within the phagosome, LLO induces pore formation to facilitate bacterial escape from host phagosomes (lysis) and aids the pathogen in intracellular replication [23]. The membrane lesions created during the process enable the passage of phospholipases which hydrolyze membrane phospholipids, resulting in complete breakdown of the plasma membrane [17]. LLO also suppresses reactive oxygen species (ROS) released by the phagocyte in response to infection, but the mechanism is yet to be understood [24]. In the cytosol, LLO prompts host histone modifications by deacetylation of H4 and dephosphorylation of Ser10 in H3 histones [25]. It also enhances infection by promoting the degradation of host proteins, though the mechanisms are yet to be understood [19]. In one study, treatment of the host proteome with LLO reduced the abundance of 149 proteins [26]. LLO causes mitochondrial fragmentation by inducing calcium influx through the ion-permeable pores on the plasma membrane [27]. However, overexpression of LLO can expose the pathogen to host immune defenses. This is a result of membrane lesions which lead to apoptosis and demolition of the pathogen’s intracellular niche, thus exposing the pathogen to the circulating defense machinery [28,29]. Host proteasomal degradation of LLO can be one mechanism employed to limit its activity within the host cytosol [28]. Another study also revealed in vitro aggregation and denaturation of LLO at 37 °C and neutral pH, suggesting that this could be another regulatory mechanism for LLO activity within the host cytosol [30].

### 2.2. Phospholipases

*L. monocytogenes* secretes phosphatidylinositol-specific phospholipase C (PI-PLC or PLC-A) encoded by the *plcA* gene and non-specific phosphotidylcholine phospholipase C (PC-PLC or PLC-B) encoded by the *plcB* gene [31]. PLC-A (PI-PLC) aids the pathogen in exiting the phagosome into the cytosol [32]. Together with PC-PLC and LLO, PI-PLC also facilitates the pathogen’s exit from the double-membrane vacuole formed upon cell-to-cell spread [33]. Both PI-PLC and PC-PLC aid *L. monocytogenes* in subverting autophagy-mediated clearance by inhibiting pre-autophagosomal maturation or target recognition by the degradative pathway [34]. Autophagy is the process through which host intracellular dysfunctional organelles are degraded and recycled [35]. Autophagy also targets invading pathogens and thus plays a role in innate immunity [35]. In one study, mutant *L. monocytogenes* strains incapable of expressing phospholipases were vulnerable to autophagy during macrophage infection [36].

PC-PLC is initially expressed as a zymogen which is then activated by a zinc metalloprotease under acidic environments [37]. It assists in vacuolar escape in times of LLO deficiency during its invasion of epithelial cells, and its activity is crucial for cell-to-cell spread [38]. PC-PLC dual enzymatic activity as a phospholipase and sphingomyelinase could be essential for *L. monocytogenes* dispersal inside the host [39]. PC-PLC is an essential pathogenicity factor contributing to *L. monocytogenes* murine cerebral listeriosis [40]. However, premature discharge of PC-PLC into the host cell cytosol can be lethal to *L. monocytogenes*; thus, the pathogen has a PC-PLC regulation mechanism for efficient pathogenicity [41]. A PC-PLC mutant exhibited low resistance against intracellular killing by neutrophils, suggesting PC-PLC may potentiate the action of neutrophils against *L. monocytogenes* [42]. *L. monocytogenes* regulates the activity of PC-PLC by increasing the pH within the double-membrane vacuole [41]. This inhibits the activity of the metalloprotease, resulting in an inactive PC-PLC [43].

### 2.3. ActA

Encoded by the *actA* gene, the protein actA enables actin recruitment and polymerization, resulting in actin-based intracellular motility in pathogenic *Listeria* species [17]. Polymerized actin close to the phagosome membrane possibly gains entry through listeriolysin-mediated pores, and its polymerization could widen the pores [44]. This results in phagosome disruption, promoting bacterial escape from the phagosome [44]. Actin propels *L. monocytogenes* to host cell membranes, resulting in elongated protrusions from the membrane (fibroids) encircling the bacteria and extending to adjacent cells which engulf the fibroids [17]. In epithelial cells, ActA camouflages the pathogen with host proteins, thus shielding it from autophagy [45]. Using a murine model, one study demonstrated that ActA is crucial for placental invasion by *L. monocytogenes* and facilitates its vertical dissemination to the fetus [46]. ActA facilitates aggregation in *L. monocytogenes* through uninterrupted ActA–ActA interactions, is involved in biofilm formation, and mediates intestinal colonization [47]. Results from one study demonstrated that actin-based intracellular motility facilitates the exit of the pathogen from autophagic membranes within the macrophage cytosol [48].

### 2.4. Internalins

Internalins are surface proteins encoded by genes which are associated with virulence in pathogenic *Listeria* species. Internalin A (InlA) and Internalin B (InlB), both encoded by the *inlAB* operon, were the first internalins to be characterized from *L. monocytogenes* [17]. E-cadherin and Met are the internalins’ receptors, respectively, both located on host cell surfaces [49]. Internalins mediate the adhesion and internalization of the pathogen by host nonphagocytic cells [50]. InlA-mediated internalization is constrained to a limited population of epithelial cells, and InlB-mediated internalization targets various cells like hepatocytes and epithelial and endothelial cells [51]. Using an epidemic strain, one study demonstrated that InlB enhances infection of the liver and spleen by *L. monocytogenes* [52].

Internalins consist of a signal peptide at the N-terminus and 22-amino acid leucin-rich-repeats (LRR) [53]. The N-terminal region alone is capable of enhancing bacterial penetration into permissive cells [54]. These LLR regions are responsible for protein–protein interactions and are present in both prokaryotic and eukaryotic cells, where they also facilitate adhesion [55]. Other pathogenic bacterial species also express LLR regions [50]. Interactions between Internalin A and E-cadherin are vital for traversing the placental and intestinal barriers [49]. The novel InlL facilitates *L. monocytogenes* attachment to abiotic surfaces and plays a role in biofilm establishment [56]. Internalin K recruits a major vault protein (MVP) that camouflages the pathogen, shielding it from autophagic recognition [57]. Other novel internalins (InlP1, InlPq, and InlP4) which contribute to the virulence of *L. monocytogenes*, have been reported [58].

## 3. Antimicrobial Resistance

### 3.1. Horizontal Gene Transfer

Listeria species are reportedly capable of exchanging resistance determinants with other bacterial species. The broad-host-range *Streptococcus agalactiae* plasmid IP501, which confers resistance against macrolides, lincosamides, chloramphenicol, and streptogramins, was reportedly transferrable to *L. monocytogenes* via conjugation [59,60]. The same plasmid was reportedly capable of replication within *Listeria* species and transference among different *Listeria* species as well as back to *Streptococcus* [61]. The antimicrobial resistance plasmid pAM Beta I (pAMβ1) was successfully transferred from *Streptococcus faecalis* to some *L. monocytogenes* isolates by conjugation [62]. In the same study, conjugates were found to replicate within the host and were transferrable among *L. monocytogenes* strains or back to *Streptococcus faecalis*. One study demonstrated conjugation of the *tet(S)* gene from *Lactococcus garvieae*, a fish pathogen, to *L. monocytogenes* [63]. The transposon Tn916 harboring the *tet(M)* gene, originally discovered in *Enterococcus faecalis*, was demonstrated to be transferrable between *Enterococcus faecalis* and *Listeria innocua* and that it was capable of jumping from one host to the other among various *Listeria* species [64]. Another Tn916-like transposon, TnFO1, harbored by multidrug-resistant *Enterococcus faecalis*, was found to be transferred from *Enterococcus. faecalis* to some species, including *Listeria innocua*, via conjugation [65].

Plasmids and transposons are thought to mediate tetracycline resistance in *Listeria* species [66]. *TetM* genes discovered in two tetracycline-resistant *L. monocytogenes* isolates were analogous to the ones previously discovered in *Staphylococcus aureus* [67]. In the same study, *TetM* genes from the other two isolates had sequences that were closely related to those of a family of conjugative transposons (Tn916-Tn1545). The Tn916-Tn1545 family is closely related to SHGII, which harbors *TetM* genes in *Enterococci*.

### 3.2. Persister Cells and Biofilm Formation

One mechanism underlying the antimicrobial resistance in *L. monocytogenes* is the formation of persister cells and biofilms. Persisters are a fraction of the overall cell population that is in a dormant, non-dividing state, which enhances them to withstand bactericidal antibiotics and harsh environmental conditions [68]. Persister cells provide an endurance strategy to *L. monocytogenes*, which is transformed from bacilli to cocci during the transition [69]. The persister phenomenon probably contributes to *L. monocytogenes* survival against disinfectants and may shield the pathogen from the host defense system for longer periods, which explains the absence of listeriosis clinical symptoms several days after infection [68]. In one study, long-term-survival cells were extremely resistant to high temperatures and pressure stresses [69]. These persisters are also able to withstand preservatives used to control heat-resistant bacteria during food processing [70].

A biofilm is a population of bacterial cells attached to each other or a surface and enclosed by a self-produced matrix of extracellular polymeric substances (EPS) (proteins, polysaccharides, and extracellular DNA) [71]. The matrix is a nutrient reservoir, facilitates adhesion, acts as a protective barrier against heat and desiccation, heavy metals, ultraviolet rays, acids, and also inhibits the permeation of antimicrobial agents by restricting their diffusion potentials [72,73]. Persistent *L. monocytogenes* strains were found to have significantly better biofilm-forming capabilities than non-persistent ones [74]. Biofilms are reportedly behind the pathogen’s increased tolerance to quaternary ammonium compounds due to changes in membrane fluidity [75]. Biofilms can also protect *L. monocytogenes* from the action of biocides, but the underlying mechanism of biocide protection is yet to be clarified [76].

### 3.3. Efflux Pumps

The minimum susceptibility of *L. monocytogenes* to antimicrobial agents is also attributed to some efflux pumps. The Lde efflux pump was found to be associated with fluoroquinolones resistance in *L. monocytogenes* isolated from listeriosis patients [77]. In the same study, the protein sequence of the Lde pump was found to be 44% similar to that of PmrA of *Streptococcus pneumoniae*, a secondary multidrug transporter of the major facilitator superfamily (MFS). Evidence from another study also suggests that the Lde efflux pump contributes to ciprofloxacin resistance in *L. monocytogenes* [78]. In the same study, minimum inhibitory concentrations (MICs) of the disinfectant benzalkonium chloride (BC) were unaffected by the absence of the *lde* gene, indicating that the Lde efflux pump may not confer BC resistance. An earlier study had reported that both the Lde and the MdrL efflux pumps may confer BC resistance to *L. monocytogenes*, alongside other mechanisms [79]. Increased *L. monocytogenes* tolerance to BC can be a result of the expression of an EmrE efflux pump [80]. The MdrL efflux pump is in charge of the extrusion of antimicrobial agents and heavy metals from the pathogen [81]. The MdrM and MdrT efflux pumps enable persistence and replication of the pathogen within the gastrointestinal tract, counteracting the bactericidal effects of mammalian bile [82]. The bile component cholic acid induces the expression of both pumps, but MdrT regulates the extrusion of cholic acid, which can be lethal to mutant strains lacking the MdrT efflux pump [83].

### 3.4. Environmental Stress Exposure

*Listeria* species may encounter sublethal concentrations of antimicrobial agents and growth inhibitors, e.g., bacteriocins and disinfectants (biocides) in the food processing and agriculture industries [84]. Such exposure may trigger adaptation to resist higher concentrations of these antimicrobials. Pre-exposure adaptation subsequently contributes to antibiotic resistance of *Listeria* species [15]. Co-selection for antibiotic resistance of *Listeria* species resulting from the use of biocides and heavy metals is reportedly a general concern [85]. One study reported significant correlations between biocide tolerance and antibiotic resistance in *Listeria* among other bacterial species isolated from seafood [86]. Unlimited exposure of the pathogen to sublethal dosages of chlorine may indirectly induce a significant tolerance to antibiotics, due to alterations in cell structure and morphology [87]. The bacteriocin nisin produced by *Lactococcus lactis* is one of the antimicrobial agents commonly applied in the food processing industry. However, nisin resistance by *L. monocytogenes* has been reported [88]. Nisin targets cell wall biosynthesis and cell membrane disruption, thus resistance may arise from alterations in the cell wall composition, hindering bacteriocin entry into the cell [89].

*L. monocytogenes* encounters environmental stresses in the food processing chain, and these stresses are usually effects of the numerous food preservation methods such as high salinity and acidic pH [90]. Adaptation of the pathogen to these sublethal stressful environmental conditions has been reported to contribute to antimicrobial resistance [91]. This is usually a result of bacterial phenotypic changes, mainly affecting cell wall and membrane structures, or alterations in efflux pump activity [92].

## 4. Environmental Stress Adaptation

### 4.1. Acidity

Acid tolerance response (ATR), arginine deiminase (ADI), and the glutamate decarboxylase (GAD) system are the three mechanisms employed by *L. monocytogenes* for persistence in low pH environments [93,94]. The ATR system is activated by pre-exposure to sublethal pH (5–6) and facilitates the pathogen’s endurance in highly acidic settings [95]. The two-component pathway also protects the pathogen from osmotic stress and high temperatures [2].

The ADI system is operated by three enzymes: arginine deiminase hydrolyzes arginine to citrulline and ammonia, ornithine carbamoyltransferase catalyzes the conversion of citrulline to ammonia and carbamoylphospate, and carbamate kinase synthesizes ATP using carbamoylphosphate and adenosine diphosphate (ADP) [94]. The GAD system promotes the survival and growth of the pathogen in mild and extremely acidic environments [96]. It also enhances its passage through the stomach (gastric environment), enabling access to and invasion of the intestinal epithelial cells. Under extremely acidic conditions, the enzyme glutamate decarboxylase (encoded by *gadA* or *gadB*) decarboxylates imported extracellular glutamic acid to γ-aminobutyrate (GABA), consuming intracellular proteins [97].

Another mechanism for acid tolerance is the F0F1–ATPase complex. The F0F1–ATPase enzyme has two different domains: a cytoplasmic catalytic portion (F_1_) responsible for ATP synthesis or hydrolysis and the integral membrane domain (F_0_), functioning as a proton channel [98]. The enzyme synthesizes ATP aerobically using protons passing into the cell cytoplasm (oxidative phosphorylation) or hydrolyzes ATP as protons exit the cell, generating a proton motive force (PMF) [99].

### 4.2. Osmotic Stress

*L. monocytogenes* is reportedly capable of withstanding high salt concentrations (NaCl) [100]. In response to osmotic stress, the pathogen activates two different mechanisms: the primary and the secondary responses. During the primary response, there is an influx of potassium cations and glutamate into the cell [89]. Uptake of osmoprotectants such as glycine betaine and carnitine follows [101]. Both mechanisms also assist the bacterium in maintaining turgidity and help in the stabilization of protein structure and function [102]. Two genes (*lmo1078* and *lmo2085*) are responsible for cell envelop modification as an adaptation to osmotic stress [103,104]. A glucose phosphorylase, *lmo1078*, synthesizes UDP-glucose, the building unit of membrane glycolipids and the cell wall [103]. *lmo2085* is an exterior protein covalently bound to cell wall peptidoglycans [104]. General stress response proteins are also expressed in response to osmotic shock, and in the absence of osmoprotectants, the protein Ctc, which confers high osmolarity resistance, is expressed [105].

### 4.3. Low Temperatures (Cold Stress)

Adaptation of *L. monocytogenes* to low temperatures involves the expression of cold shock proteins (CSPs) [106]. These are small proteins which assume the role of molecular chaperones, enabling replication, transcription, translation, and protein folding at low temperatures [107]. Another adaptation mechanism is the importation of glycine betaine and carnitine as cryoprotectants [90]. Increased intracellular solutes levels help reduce the loss of intracellular water in times of low temperatures [89]. In one investigation, a mutant *L. monocytogenes* strain for glycine betaine and carnitine uptake systems failed to accumulate or transport any of the two and, thus, it could not survive at low temperatures [108]. *L. monocytogenes* also alter its membrane structure at low temperatures. The membrane’s rigidity increases due to an upsurge in the concentration of unsaturated fatty acids [109]. This prevents the development of a gel-like structure which may allow leakage of the cytoplasmic content [110].

### 4.4. Oxidative Stress

Oxidative stress arises from exposure to ROS (superoxide, hydroxyl radicals, and hydrogen peroxide) which may be generated during food processing [111]. Adaptations to oxidative stress includes ROS detoxification systems such as catalase (Cat), superoxide dismutase (Sod), and alkyl hydroperoxidase (AhpCF) [112]. Ferritin-like protein (*fri*) reportedly protects *L. monocytogenes* from the oxidative effects of hydrogen peroxide [113].

## 5. Conclusions

*L. monocytogenes* undoubtedly remains a foodborne pathogen of public health concern, owing to the high mortality and hospitalization rates caused by its infection [73]. The 2017–2018 listeriosis outbreak in South Africa was a clear evidence that, despite the development of treatments and biocontrol agents to combat *L. monocytogenes*, the pathogen remains a threat to food safety [114]. This is further worsened by the increasing tolerance of the pathogen to biocontrol agents and antimicrobials. Environmental stresses also have an impact on the pathogen’s physiology, morphology, pathogenicity, gene expression, and antimicrobial resistance [91]. Understanding the pathogen’s virulence, antimicrobial resistance mechanisms, and environmental stress adaptation will significantly contribute to the development of novel efficient, cost-effective antimicrobial agents and biocontrol methods for combating the pathogen. Continuous monitoring, stringent surveillance, and source tracking are also crucial for the detection of the pathogen’s antimicrobial resistance developments [15].
